# Alcohol brief intervention delivered in UK community pharmacies: customers’ experiences

**DOI:** 10.1186/1940-0640-7-S1-A23

**Published:** 2012-10-09

**Authors:** Cate Whittlesea, Ranjita Dhital, Ian Norman

**Affiliations:** 1 Institute of Pharmaceutical Science, King's College London, London, UK; 2 Division of Health and Social Care Research, King's College London, London, UK

## 

Pharmacy-based alcohol brief intervention (BI) has the potential to identify risky drinkers in the general population, but the opinions of users regarding the service remain relatively unexplored. Alcohol BI was offered to customers by trained pharmacists (n = 29) at 28 London, UK, community pharmacies between February and July 2010. Customers requiring alcohol-use related medication and/or advice were targeted. The Alcohol Use Disorders Identification Test-Consumption (AUDIT-C), a drinking diary, and a readiness to change assessment were used by pharmacists to assess and provide appropriate feedback regarding alcohol use. Customers also received written information, including a UK Department of Health “Units and You” booklet. Following BI, customers were given a confidential service evaluation questionnaire to complete and return to the project team using a prepaid envelope. This invited responses to closed- and open-format questions regarding their initial reason for visiting the pharmacy, why they took up the BI service, and their levels of satisfaction with the service delivery and environment. Of the 134 customers who received a BI, 58% (n = 78) returned the questionnaire. Bringing a prescription for dispensing was the most common reason for the pharmacy visit (55%, n = 43). Wishing to find out about alcohol use and concerns for personal health were the two most reported reasons for taking up the service. Almost one-quarter of customers (n = 18) reported that they liked having increased their alcohol-related awareness, and 18% (n = 14) indicated that they liked the informative written information. The privacy (74%, n = 57), confidentiality (77%, n = 59), and quietness (70%, n = 54) of consulting rooms was rated as good, with 77% (n = 60) of customers reporting they would recommend this service to others. In line with past primary care BI studies, customers were generally positive about the experience of receiving BI in community pharmacies and would recommend it to others.

